# Supplementary material for: Dynalign II: common secondary structure prediction for RNA homologs with domain insertions
Source: Nucleic Acids Res. 2014 Nov 21;42(22):13939–48. doi: 10.1093/nar/gku1172 (PMC4267632; doi:10.1093/nar/gku1172)
Supplement: SUPPLEMENTARY DATA [file supp_gku1172_nar-02021-z-2014-File012.zip › manual/javadoc/RNAstructure_java_drawing/source/proxy/StructureBackendProxyJNI.html]

StructureBackendProxyJNI


JavaScript is disabled on your browser.


- Overview
- Package
- Class
- Tree
- Index
- Help

- Prev Class
- Next Class

- Frames
- No Frames

- All Classes

- Summary:
- Nested |
- Field |
- Constr |
- Method

- Detail:
- Field |
- Constr |
- Method


RNAstructure\_java\_drawing.source.proxy

## Class StructureBackendProxyJNI

- java.lang.Object
- - RNAstructure\_java\_drawing.source.proxy.StructureBackendProxyJNI

- ---

    

  ```
   class StructureBackendProxyJNI
  extends Object
  ```

- - ### Constructor Summary

    Constructors

    Constructor and Description

    `StructureBackendProxyJNI()`
  - ### Method Summary

    Methods

    | Modifier and Type | Method and Description |
    | --- | --- |
    | `static void` | `delete_StructureBackend(long jarg1)` |
    | `static long` | `new_StructureBackend()` |
    | `static boolean` | `StructureBackend_addAnnotationProbability(long jarg1, StructureBackend jarg1_, String jarg2)` |
    | `static boolean` | `StructureBackend_addAnnotationSHAPE(long jarg1, StructureBackend jarg1_, String jarg2)` |
    | `static void` | `StructureBackend_flip(long jarg1, StructureBackend jarg1_)` |
    | `static String` | `StructureBackend_getStructureData(long jarg1, StructureBackend jarg1_, int jarg2)` |
    | `static boolean` | `StructureBackend_readStructureData(long jarg1, StructureBackend jarg1_, String jarg2)` |
    | `static void` | `StructureBackend_removeAnnotation(long jarg1, StructureBackend jarg1_)` |
    | `static void` | `StructureBackend_setNucleotidesCircled(long jarg1, StructureBackend jarg1_, boolean jarg2)` |
    | `static String` | `StructureBackend_writeDotBracketFile(long jarg1, StructureBackend jarg1_, String jarg2, String jarg3)` |
    | `static String` | `StructureBackend_writeHelixFile(long jarg1, StructureBackend jarg1_, String jarg2, String jarg3, int jarg4)` |
    | `static void` | `StructureBackend_writePostscriptFile(long jarg1, StructureBackend jarg1_, String jarg2, int jarg3)` |
    | `static void` | `StructureBackend_writeSVGFile(long jarg1, StructureBackend jarg1_, String jarg2, int jarg3)` |

    - ### Methods inherited from class java.lang.Object

      `clone, equals, finalize, getClass, hashCode, notify, notifyAll, toString, wait, wait, wait`

- - ### Constructor Detail


    - #### StructureBackendProxyJNI

      ```
      StructureBackendProxyJNI()
      ```
  - ### Method Detail


    - #### delete\_StructureBackend

      ```
      public static final void delete_StructureBackend(long jarg1)
      ```


    - #### new\_StructureBackend

      ```
      public static final long new_StructureBackend()
      ```


    - #### StructureBackend\_addAnnotationProbability

      ```
      public static final boolean StructureBackend_addAnnotationProbability(long jarg1,
                                                      StructureBackend jarg1_,
                                                      String jarg2)
      ```


    - #### StructureBackend\_addAnnotationSHAPE

      ```
      public static final boolean StructureBackend_addAnnotationSHAPE(long jarg1,
                                                StructureBackend jarg1_,
                                                String jarg2)
      ```


    - #### StructureBackend\_flip

      ```
      public static final void StructureBackend_flip(long jarg1,
                               StructureBackend jarg1_)
      ```


    - #### StructureBackend\_getStructureData

      ```
      public static final String StructureBackend_getStructureData(long jarg1,
                                             StructureBackend jarg1_,
                                             int jarg2)
      ```


    - #### StructureBackend\_readStructureData

      ```
      public static final boolean StructureBackend_readStructureData(long jarg1,
                                               StructureBackend jarg1_,
                                               String jarg2)
      ```


    - #### StructureBackend\_removeAnnotation

      ```
      public static final void StructureBackend_removeAnnotation(long jarg1,
                                           StructureBackend jarg1_)
      ```


    - #### StructureBackend\_setNucleotidesCircled

      ```
      public static final void StructureBackend_setNucleotidesCircled(long jarg1,
                                                StructureBackend jarg1_,
                                                boolean jarg2)
      ```


    - #### StructureBackend\_writeDotBracketFile

      ```
      public static final String StructureBackend_writeDotBracketFile(long jarg1,
                                                StructureBackend jarg1_,
                                                String jarg2,
                                                String jarg3)
      ```


    - #### StructureBackend\_writeHelixFile

      ```
      public static final String StructureBackend_writeHelixFile(long jarg1,
                                           StructureBackend jarg1_,
                                           String jarg2,
                                           String jarg3,
                                           int jarg4)
      ```


    - #### StructureBackend\_writePostscriptFile

      ```
      public static final void StructureBackend_writePostscriptFile(long jarg1,
                                              StructureBackend jarg1_,
                                              String jarg2,
                                              int jarg3)
      ```


    - #### StructureBackend\_writeSVGFile

      ```
      public static final void StructureBackend_writeSVGFile(long jarg1,
                                       StructureBackend jarg1_,
                                       String jarg2,
                                       int jarg3)
      ```


- Overview
- Package
- Class
- Tree
- Index
- Help

- Prev Class
- Next Class

- Frames
- No Frames

- All Classes

- Summary:
- Nested |
- Field |
- Constr |
- Method

- Detail:
- Field |
- Constr |
- Method
